# DNA Damage in Nijmegen Breakage Syndrome Cells Leads to PARP Hyperactivation and Increased Oxidative Stress

**DOI:** 10.1371/journal.pgen.1002557

**Published:** 2012-03-01

**Authors:** Harald Krenzlin, Ilja Demuth, Bastian Salewsky, Petra Wessendorf, Kathrin Weidele, Alexander Bürkle, Martin Digweed

**Affiliations:** 1 Institute of Medical and Human Genetics, Charité – Universitätsmedizin Berlin, Berlin, Germany; 2 The Berlin Aging Study II, Research Group on Geriatrics, Charité – Universitätsmedizin Berlin, Berlin, Germany; 3 Molecular Toxicology, Department of Biology, University of Konstanz, Konstanz, Germany; The University of North Carolina at Chapel Hill, United States of America

## Abstract

Nijmegen Breakage Syndrome (NBS), an autosomal recessive genetic instability syndrome, is caused by hypomorphic mutation of the *NBN* gene, which codes for the protein nibrin. Nibrin is an integral member of the MRE11/RAD50/NBN (MRN) complex essential for processing DNA double-strand breaks. Cardinal features of NBS are immunodeficiency and an extremely high incidence of hematological malignancies. Recent studies in conditional null mutant mice have indicated disturbances in redox homeostasis due to impaired DSB processing. Clearly this could contribute to DNA damage, chromosomal instability, and cancer occurrence. Here we show, in the complete absence of nibrin in null mutant mouse cells, high levels of reactive oxygen species several hours after exposure to a mutagen. We show further that NBS patient cells, which unlike mouse null mutant cells have a truncated nibrin protein, also have high levels of reactive oxygen after DNA damage and that this increased oxidative stress is caused by depletion of NAD^+^ due to hyperactivation of the strand-break sensor, Poly(ADP-ribose) polymerase. Both hyperactivation of Poly(ADP-ribose) polymerase and increased ROS levels were reversed by use of a specific Poly(ADP-ribose) polymerase inhibitor. The extremely high incidence of malignancy among NBS patients is the result of the combination of a primary DSB repair deficiency with secondary oxidative DNA damage.

## Author Summary

Damage to DNA is extremely dangerous because it can lead to mutations in genes that initiate or accelerate the development of a tumor. Evolution has led to highly complex networks of DNA repair enzymes, which for the majority of individuals are extremely effective in keeping our DNA intact. The devastating consequences of DNA damage are manifested in those individuals in which one or other of the repair pathways is non-functional. Several genetic disorders can be attributed to such DNA repair deficiencies and have the common feature of increased tumor incidence as the major life-threatening symptom. Cancer incidence varies amongst these disorders and is probably highest for the disease Nijmegen Breakage Syndrome, where more than 50% of patients develop a hematological malignancy in childhood. We have sought to understand this extremely high incidence by exploiting cells from a mouse model and cells derived from patients. We find that deficiency in the repair of DNA double-strand breaks leads to disturbances in cellular metabolism, leading ultimately to a loss of antioxidative capacity. The ensuing accumulation of highly reactive oxygen species generates further DNA lesions, thus potentiating the initial damage and increasing the likelihood of malignancy.

## Introduction

Genetic cancer susceptibility disorders, such as Xeroderma pigmentosum and Fanconi anemia, generally have deficiencies in DNA repair and cell cycle regulation leading to tumour initiation. The specific mutagen sensitivities underlying these disorders define a set of enzymes and pathways involved in the DNA damage response. Nevertheless, these pathways clearly overlap and components in one pathway can be critically involved in another. For example, the nucleotide excision repair pathway mutated in Xeroderma pigmentosum is also required for the repair of interstrand crosslinks, to which Fanconi anemia patient cells are particularly sensitive [1].

Nijmegen Breakage Syndrome (NBS), Nijmegen Breakage Syndrome like disorder (NBSLD), Ataxia telangiectasia (AT) and Ataxia telangiectasia like disorder (ATLD) are clinically and biologically overlapping entities. Whilst the underlying proteins are intimately associated, cancer predisposition is a major life threatening feature of NBS and AT only. The proteins mutated in these four disorders are all involved in the sensing and repair of DNA double-strand breaks (DSB). However, if, as seems likely, the mutation rate in patient cells is increased, this may not be solely due to the primary DNA lesion but, rather, to the cumulative effects of auxiliary cellular disturbances. Thus it has been repeatedly shown that AT patient cells and knockout mice have increased oxidative stress [2]–[4] which could contribute to clinical progression of the disease.

Oxidative stress has not previously been associated with NBS, however, our previous proteomic study of null mutant mice suggested disturbances in the redox homeostasis in the livers of irradiated mice [5]. We speculated that this could be due to hyperactivation of members of the Poly(ADP-ribose) polymerase (PARP) family, such as PARP-1, PARP-2 and PARP-3 which rapidly detect DNA strand breaks and regulate/modulate proteins required for an effective cellular response. In cells unable to repair DSBs, the permanent activation or even hyperactivation of PARP enzymes was expected to disturb cellular function and contribute to an increased mutation rate. Interestingly, poly(ADP-ribosyl)ation was reported to be unaffected in both AT patient cells and knock out mouse cells [6]. In view of the close relationship between NBS and AT we sought to examine the situation in NBS. For these investigations we have started with our null mutant mouse cells since, unlike patient cells, they provide a system with complete absence of the affected protein, nibrin.

We find greatly increased levels of reactive oxygen species in both null mutant mouse cells and NBS patient cells after a DNA damaging exposure. Unlike AT cells, we find a parallel increase in the activity of PARP enzymes as measured by examining poly(ADP-ribosyl)ation of proteins. As we have previously hypothesized, depletion of the cellular NAD^+^ pool accompanies excessive poly(ADP-ribosyl)ation in NBS cells and this severely compromises the anti-oxidant capacity of the cells. Thus the extremely high incidence of hematological malignancies in NBS may be the result of the combination of a primary DSB repair deficiency and accompanying oxidative damage.

## Results

### Increased reactive oxygen species after DNA damage in *Nbn* null mutant murine fibroblasts and NBS patient cells

**Figure 1:**
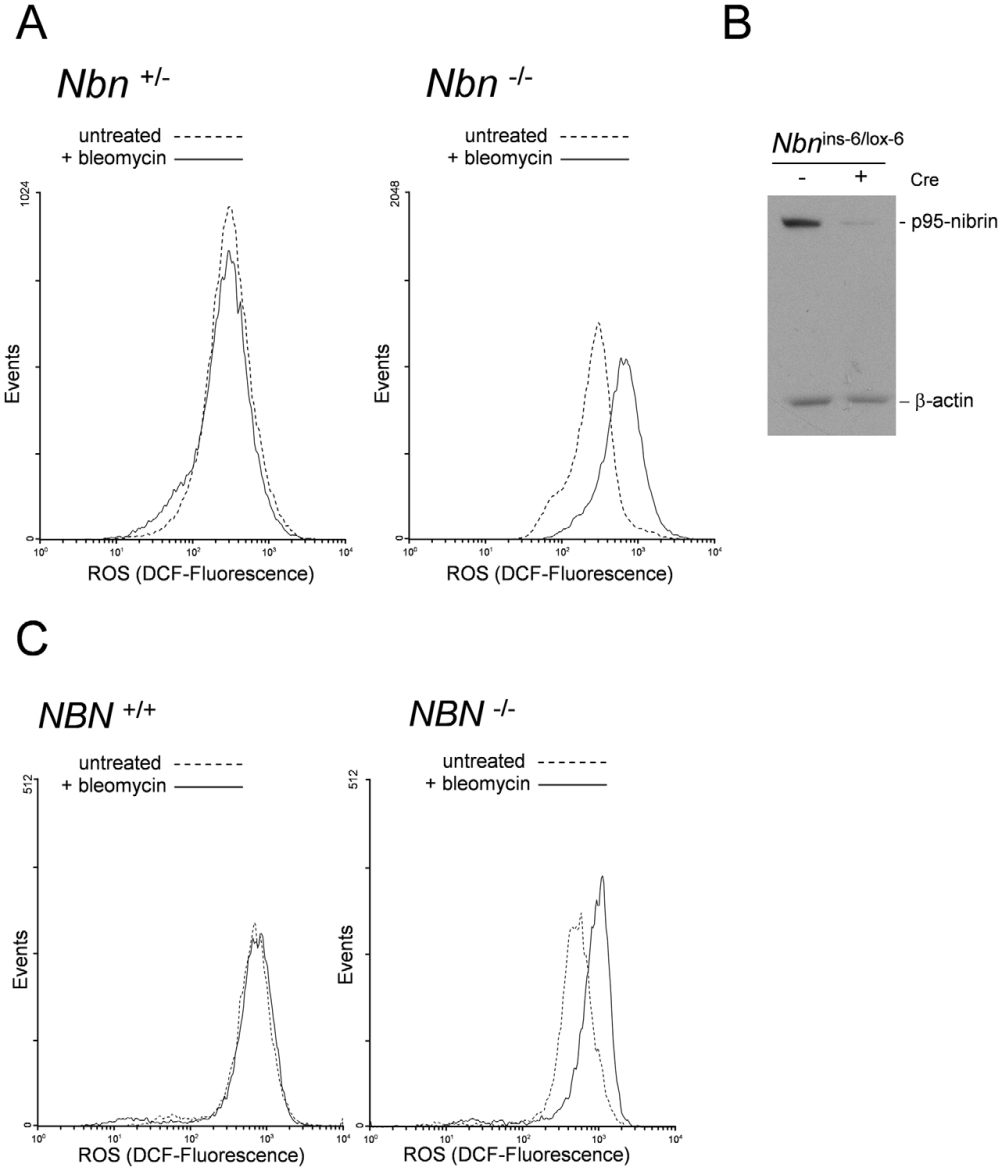
High levels of ROS in *Nbn* null mutant murine fibroblasts and NBS patient cells after DNA damage. (A) FACS profiles of ROS measurements in murine cells with the indicated genotypes with or without treatment with bleomycin. Cells were stained with CM-H_2_DCFDA 12 hours after treatment with bleomycin. Fluorescence intensity is proportional to ROS. The experiment was repeated six times and the same profiles were obtained. (B) Western-blot demonstration of conditional *Nbn* null mutation in murine fibroblasts. Lysates from *Nbn* ^Ins-6/lox-6^ fibroblasts with and without treatment with HTNC were probed on immunoblots with anti-nibrin and anti-actin antibodies. (C) Representative FACS profiles of ROS measurements in LN9 wild type and GM7166VA7 NBS patient fibroblasts with or without treatment with bleomycin. Cells were stained with CM-H_2_DCFDA 12 hours after treatment with bleomycin. Fluorescence intensity is proportional to ROS. The experiment was repeated more than five times and essentially the same profiles were obtained.

The murine fibroblasts used in these experiments have a neomycin insertion in one *Nbn* allele (*Nbn* ^ins-6^), a null mutation, and loxP sites flanking exon six in the other *Nbn* allele (*Nbn* ^lox-6^). Treatment of these cells with cre recombinase leads to cells with biallelic *Nbn* ^ins-6/del6^ null mutations [7]. Henceforth we refer to wild type alleles and alleles with exon 6 flanked by loxP sites as *Nbn* ^+^ and the null mutant *Nbn* ^ins-6^ and *Nbn* ^del-6^ alleles as *Nbn* ^−^. As shown in Figure 1A, 12 hours after introduction of DSBs there is a particularly high level of ROS in fibroblasts completely lacking nibrin due to null mutation of the *Nbn* gene (Figure 1B). The cells were treated here with 10 µg/ml bleomycin, which is equivalent to irradiation with 2 Gy irradiation causing approximately 60 DSBs per cell, with a ratio of DSBs to single-strand breaks of 1∶9 [8], [9]. As the non-fluorescent compound, CM-H_2_DCFDA, is converted to fluorescein specifically by hydrogen peroxide, hydroxyl radicals, peroxynitrite anion and peroxyl radicals, the observed over two-fold increase in fluorescence intensity in comparison to heterozygous cells is therefore due to the accumulation of these species [10]–[12]. These radicals are short lived with half-lives of just seconds or less [13], [14]. Therefore, their high concentration 12 hours after treatment with bleomycin suggests that they are being permanently produced in the *Nbn^−/−^* cell, presumably as a consequence of its unrepaired DSBs.

The null mutant murine cells examined here are particularly useful since they allow examination of cellular responses in the complete absence of nibrin, a situation not naturally available for human cells. Having seen the importance of full length nibrin for maintenance of cellular redox homeostasis by timely repair of DSBs, we turned to NBS patient cells, in which a truncated and partially functional nibrin fragment, p70-nibrin, is present [15], [16]. As shown in Figure 1C, fibroblasts from NBS patients also show an increased level of ROS after DNA damage. The increase in ROS-induced fluorescence, 1.5(+/−0.27) times that of controls, is less than in the complete absence of nibrin, 2.33(+/−0.9) times, which might indicate partial repair of DSBs or simply reflect differences in murine and human cells in ROS induction.

**Figure 2:**
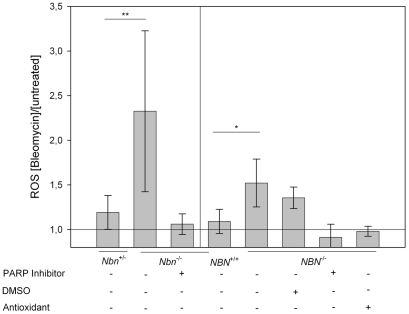
ROS in *Nbn* null mutant murine fibroblasts and NBS patient cells after DNA damage. Relative levels of ROS after treatment with bleomycin are given for murine and human LN9, GM166VA7 and NBS-1LBI cells with the given genotypes and after the indicated treatments. ** p = 0.0095 in the Mann-Whitney U Test (two-tailed, n^1^ = 4, n^2^ = 6); * p = 0.017 in the Mann-Whitney U Test (two-tailed, n^1^ = 3, n^2^ = 12).

The results of repeated measurements of ROS levels in *Nbn* null mutant and NBS patient cells are shown in Figure 2. As indicated in the figure, the differences in ROS levels in comparison to wild type cells after DNA damage are statistically significant in the non-parametric two-tailed Mann-Whitney test.

### Hyperactivation of Poly(ADP-ribose) polymerases in the absence of full-length nibrin

**Figure 3:**
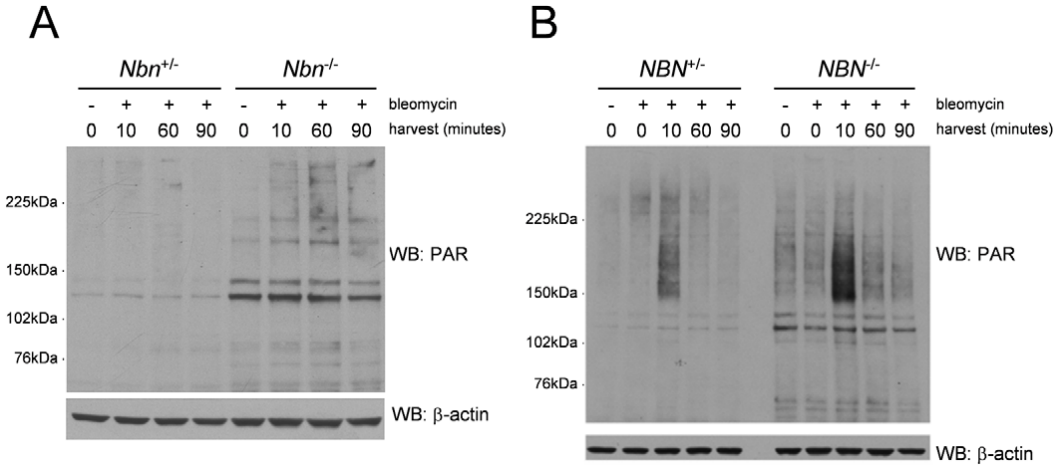
Increased PARP activity in *Nbn^−/−^* murine fibroblasts and NBS patient fibroblasts after DNA damage. Lysates from mouse (A) and human LN9 and GM166VA7 fibroblasts (B) with the given genotypes were harvested at the indicated timepoints (minutes) after a bleomycin treatment and probed on immunoblots with antibodies directed against poly(ADP-ribose) and ß-actin.

We have argued previously that the permanent production of ROS in the absence of nibrin is caused by rapid depletion of NAD^+^ due to hyperactivation of Poly(ADP-ribose) polymerases and consequent loss of cellular antioxidant capacity [5]. In order to test this hypothesis, we treated cells heterozygous and homozygous for *Nbn* null mutations with bleomycin to induce DSBs and examined the extent and kinetics of poly(ADP-ribosyl)ation by western blot. As shown in Figure 3A, there is rapid and sustained poly(ADP-ribosyl)ation of proteins in the absence of nibrin under conditions in which PARP enzyme activity in heterozygous cells cannot be detected.

In Figure 3B PARP activity is shown for control fibroblasts and fibroblasts from NBS patients. Even in these cells with a partially active nibrin fragment, there is rapid and extensive activation of PARP as evidenced by poly(ADP-ribosyl)ation of proteins. By densitometric analysis of three independent blots we found a 3-fold increase in PAR-modified proteins in control fibroblasts 10 minutes after a DNA damaging treatment and a 17-fold increase in NBS patient fibroblasts (p = 0.05). Levels of protein poly(ADP-ribosyl)ation have returned to near normal 12 hours after treatment (data not shown).

**Figure 4:**
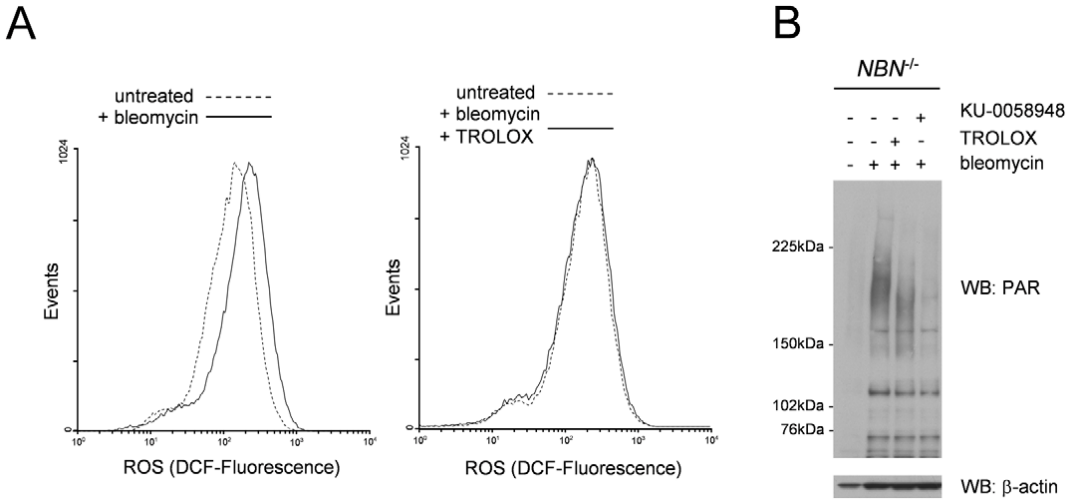
ROS levels in NBS patient fibroblasts after DNA damage are reduced by antioxidant scavengers but PARP remains hyperactivated. (A) FACS profiles of ROS measurements in NBS-1LBI patient cells 12 hours after treatment with bleomycin and in the presence or absence of the antioxidant TROLOX. Cells were stained with CM-H_2_DCFDA for ROS detection. The data shown are from one of three experiments with essentially identical results. (B) Lysates from NBS-1LBI patient cells were harvested 15 minutes after DNA damage by bleomycin in the presence the PARP inhibitor KU-0058948 or the antioxidant TROLOX as indicated. Lysates were probed on immunoblots with antibodies directed against poly(ADP-ribose) and ß-actin.

We reasoned that if the increased ROS levels in NBS patient cells are a consequence of increased PARP activity, rather than its cause, inhibition of the enzyme should reduce ROS levels, whilst scavenging of ROS should not affect PARP activity. As shown in Figure 4, scavenging ROS using the antioxidant vitamin E derivative TROLOX reduced ROS levels in damaged NBS patient cells to the same levels as in untreated cells (Figure 4A and Figure 2). However, although cells treated with TROLOX and bleomycin were essentially ROS free, PARP activity remained high (Figure 4B). Inhibition of PARP-1, PARP-2 and PARP-3 with the specific inhibitor KU-0058948 [17], on the other hand (Figure 4B), did reduce ROS levels to normal (Figure 2).

### Rapid depletion of NAD^+^ in NBS patient cells after DNA damage

**Figure 5:**
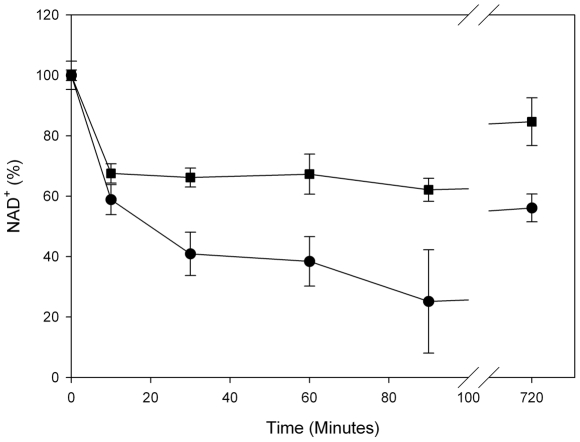
Rapid NAD^+^ depletion in NBS patient fibroblasts after DNA damage. Relative levels of NAD^+^ in NBS-1LBI NBS patient fibroblasts (•) and LN9 control fibroblasts (▪) after DNA damage are shown. NAD^+^ levels in untreated cells were set at 100%.

The link between ROS levels and PARP enzyme activity is the latter's requirement for NAD^+^, an important component of the cells antioxidant capacity. Indeed, numerous reports have shown that PARP inhibition prevents the reduction of NAD^+^ concentrations in cells subject to genotoxins, with a resulting decrease in cellular necrosis [18], [19]. As shown in Figure 5, we measured NAD^+^ levels in NBS fibroblasts after a bleomycin treatment in comparison to normal fibroblasts. There is very rapid depletion of NAD^+^ in the patient cells in comparison to the control cells, confirming the hyperactivation of PARP in these cells. Interestingly, the baseline levels of NAD^+^ in these NBS patient cells were considerably higher than in controls (1,850(+/−86) vs. 875(+/−15) pmol/10^6^ cells) suggesting that even in the absence of exogenous damaging agents, NAD^+^ requirements are higher in these repair deficient cells. In line with this observation we note that PARP activity in undamaged NBS cells is apparently higher than in controls (Figure 3B). Even 12 hours after treatment with bleomycin, baseline levels of NAD^+^ have still not been reached in NBS cells, in agreement with the timing of ROS measurements shown in Figure 1 and Figure 2.

## Discussion

Nibrin is a component of the trimeric MRN complex together with Mre11 and RAD50. This complex is involved in the processing of all DNA double-strand breaks in the cell, whatever their origin: mutagen exposure, physiological processes or simply chromosome ends [20]. The complex is implicated in DSB repair by both non-homologous end joining and homologous recombination [21], [22]. As a sensor of DSBs the MRN complex is involved in the activation of ATM and subsequent downstream targets to induce cell cycle checkpoints [23]. Telomeres, the ends of chromosomes, are maintained by a mechanism in which the MRN complex has also been implicated [24].

Cancer incidence in Nijmegen Breakage Syndrome is extremely high with 40% of patients developing a tumor, mostly lymphoma, before the age of 20 [25], [26]. This contrasts with the related disorder AT in which lifetime cancer risk is 20–30% [27], [28]. For accurate prognosis and improved patient care it is important to establish which factors contribute to this cancer predisposition. In this respect, the role of nibrin in both DNA repair and cell cycle regulation may be significant. It has been shown that mutation of *Nbn* in mouse models leads to a defect in apoptosis [29], [30], [31] and reduced clearance of damaged cells could clearly contribute to the high cancer incidence [32]. In addition, factors leading to an increased mutation rate, beyond that of the primary double strand break, could be present in NBS. Previous work has suggested that oxidative stress could be one such factor [5].

Unphysiologically high levels of ROS are a hallmark of oxidative stress and can be directly due to mutagenic agents, such as ionizing radiation, or, rather, reflect overburden of the cellular antioxidation mechanisms. These mechanisms can be either direct scavenging of radicals or regeneration of oxidized biomolecules [33]. NADPH and NADH have been reported to be involved in both kinds of antioxidant activity [34]. Thus, reduction in the availability of these essential cellular antioxidants leads inevitably to increased cellular ROS levels and oxidative stress, even in the absence of DNA damage. The radicals detected in this report, peroxynitrite anion, hydroxyl and peroxyl radicals or their metabolites, all react aggressively with DNA to yield oxidized bases and single strand breaks. An increased mutation rate would be the consequence. In lymphocytes, in which DSBs are a prerequisite for immunoglobulin gene rearrangements, their non-repair due to the absence of nibrin could thus lead to redox disturbances and an even higher occurrence of mutations.

NAD^+^ is the precursor for NAD(P)H and its cellular level is therefore critical for cellular redox homeostasis. NAD^+^ is also a substrate for the PARP superfamily of enzymes with a common catalytic activity and involved in the DNA damage response [35], [36]. For example, PARP-1 is a nuclear DNA damage sensor and binds to persisting single- and double-strand breaks [37]. PARP enzymes covalently attach ADP-ribose to glutamate, aspartate, and lysine residues of acceptor proteins. Branched ADP-ribose polymers are formed at nuclear acceptor proteins that facilitate DNA repair through modifying and activating structural proteins and enzymes such as histone H2AX, topoisomerase I and II, DNA polymerase α and β, DNA ligase I and II, nuclear factor (NF)-κB, and p53. Hyperactivation of PARP has been frequently described in various systems leading to depletion of the NAD^+^ pool [38]–[40]. It has also been suggested as a contributing factor in AT [41]. The unrepaired DSBs in *Nbn* null mutant cells clearly lead to such PARP hyperactivation, as shown here. As previously reported, the *Nbn^−/−^* cells attempt to combat the increased ROS levels due to NAD^+^ depletion by upregulating genes involved in the detoxification of radicals, such as MnSOD. In contrast, enzymes also requiring the NAD^+^ substrate were downregulated, for example, glyoxylate reductase 6.7-fold [5].

In *Nbn^−/−^* cells and also in NBS patient cells, the loss of full nibrin function leads to a delay in the activation of ATM [42]. It has recently been shown that ATM, in addition to its direct role in the DNA damage response, also promotes the pentose phosphate pathway leading to increased NADPH levels and thus improving anti-oxidant defence [43]. In the absence of nibrin, promotion of the pentose phosphate pathway will not occur, indeed in our proteomics analysis of irradiated *Nbn^−/−^* mouse livers, transaldolase, a key enzyme of the pentose phosphate pathway, was actually reduced 6-fold [5].

Human cells with null mutation of the *NBN* gene are non-viable and NBS patients all have hypomorphic mutations and express a truncated nibrin protein [15], [44]. In the case of the major founder mutation, c.657_661del5 (p.K219fsX19), the truncated protein, p70-nibrin, is translated from an upstream start codon brought into frame by the deletion [15]. These proteins clearly have enough partial activity to ensure survival [7], [45], but are severely compromised in the DNA damage response. This is manifest as the increased chromosome breakage, characteristic translocations, radiosensitivity, immunodeficiency and cancer predisposition characteristic of NBS [25]. These partially active proteins all have the carboxy terminal MRE11 and ATM interacting domains but lack the FHA and first BRCT domains of the amino-terminus, which are required for interaction with proteins such as gamma-H2AX, MDC1 and p53BP1 [46], [47], [48].

Here we describe increased ROS levels after DNA damage in NBS patient cells. The truncated p70-nibrin is clearly unable to fully prevent the hyperactivation of PARP, NAD^+^ depletion and ROS generation. Patient cells showed approximately 1.5 times the level of ROS after DNA damage in comparison to control cells whilst in null mutant mouse cells the levels were more than two fold increased. We have previously described individual variations in the level of p70-nibrin expression [16] which are due to differences in its proteasomal degradation [49]. Low levels of p70-nibrin correlate with cancer incidence and it can be speculated that a contributing factor may be higher oxidative stress.

In conclusion we present evidence for a further detrimental consequence of NBN mutation. In addition to a DSB repair deficiency and failure in cell cycle checkpoints, lack of fully functional nibrin results in increased ROS levels and oxidative stress. This unique combination would lead to an extremely high mutation rate in cells with an underlying apoptosis deficiency. Oncogene activation and tumour initiation are the consequence.

## Materials and Methods

### Cell culture, cre recombinase, and mutagen treatment

Spontaneous transformed murine fibroblasts were grown from ear explants of *Nbn* ^lox-6/ins-6^ mice [7]. Cells were cultured in Dulbecco's modified Eagle's medium (DMEM; Gibco, Life technologies) supplemented with 5% glucose (glc) and 10% fetal calf serum (FCS) strictly in the absence of antibiotics. Cell culture conditions were 37°C and 5% CO_2_. Environmental oxygen was reduced to 10%. Cells were split 1∶10 at least twice a week.

The immortalized human NBS cell lines GM7166VA7 and NBS-1LBI homozygous for *NBN* ^657del5/657del5^ and a control cell line, LN9, transformed with simian virus 40 (SV40) were cultured using the same conditions described above.

The cre recombinase fusion protein, HTNC [50], was isolated as previously described [7]. Exponentially growing cells were incubated in 2 µM HTNC twice for 6 hours in a 48 hour period. Knock-down efficiency was verified by western blot analysis using a polyclonal rabbit antibody to detect murine nibrin (Pineda Antikörper-Service, Berlin). Murine polyclonal antibody against β-actin served as a loading control (Abcam).

DNA damage was induced by incubating cells for two hours in the radiomimetic drug bleomycin at 10 µg/ml. Cells were then washed with medium and returned to culture for the specified times.

### PAR detection and Western blot

Cells were scraped into ice-cold PBS, pelleted and snap frozen in liquid nitrogen. Cell pellets were stored at −80°C. For analysis, pellets were solubilised in LDS-sample buffer (Invitrogen) and sonified for 60 seconds using a model 450 sonifier (Branson, Emerson Industrial Automation). Proteins were separated on Tris-Acetate gels (3–8%) and transferred to PVDF membranes (Hybond-P, GE Healthcare).

To detect poly(ADP-ribosyl)ated proteins, two different polyclonal antibodies (Abcam) each producing the characteristic smear of PAR modified proteins were used. As a loading control β-actin was detected using a murine polyclonal antibody. Blots were repeated three times using independent lysates. For densitometry, films were scanned using the ScanMaker scanner (Mikrotek) and lanes quantified using ImageQuant software (Molecular Dynamics).

### PARP inhibition and ROS scavenging

The PARP inhibitor KU-0058948 was kindly provided by KuDOS Pharmaceuticals Ltd. (AstraZeneca PLC). The compound was dissolved in 100% dimethylsulfoxid (DMSO) and stored at −20°C. Cells were treated with 1 µM inhibitor in medium containing 0.5% DMSO for 10 hours before induction of DNA damage and then for a further 12 hours. Control cells were incubated in parallel in medium containing 0.5% DMSO.

In some experiments, ROS were scavenged by treating cells with the antioxidant vitamin E derivative, 6-hydroxy-2,5,7,8-tetramethylchroman-2-carboxylic acid (TROLOX, Hoffman-La Roche). Cells were incubated in 500 µM TROLOX for 12 hours after the bleomycin damaging treatment.

### Quantification of intracellular reactive oxygen species

The amount of intracellular ROS was monitored before and after the induction of DNA damage in fibroblasts at 50% confluence. Cells were washed and harvested into PBS and 10^6^ cells were stained in 500 µl PBS with 10 mM 5-(and-6)-chloromethyl-29,79-dichlorodihydrofluorescein diacetate (CM-H_2_DCFDA; Invitrogen) for 20 min at 37°C in the dark. Samples were subsequently washed using ice-cold PBS and centrifuged for 10 min at 1000 rpm (∼180×g) before being resuspended in FACS dissociation solution (FACSmax, Genlantis) and kept on ice until analysis. Flowcytometry was performed using the FACS-Calibur (Becton Dickinson Bioscience) counting a minimum of 10^4^ cells per sample. The opensource flowcytometry software WinMDI V2.9 was used for data analysis. Gates were placed on dot blots of forward vs. side scatter to exclude apoptotic cells and debris from the fluorescence histograms shown in the figure. In all measurements at least 85% of cells were within this gate. All experiments were repeated independently at least three times. Relative ROS-levels are expressed as [gated mean of bleomycin treated cells]/[gated mean of untreated cells] and were evaluated for statistical significance using the non parametric two-tailed Mann-Whitney U test.

### Quantification of NAD^+^ levels in fibroblasts

NBS patient fibroblasts and control fibroblasts were treated in triplicate with bleomycin as indicated above. At the timepoints indicated in Figure 5 cells were precipitated with 0.5 M perchloric acid on ice. After 15 min samples were centrifuged at 1500×g for 10 min and the supernatant (500 µl) was combined with 350 µl of 1 M KOH, 0.33 M K2HP04, 0.33 M KH2P04 followed by incubation on ice for 15 min.

Cells were centrifuged at 1500×g for 10 min and the supernatant was frozen at −20°C before NAD^+^ determination by using an enzymatic cycling assay [51].
